# Understanding collaborative implementation between community and academic partners in a complex intervention: a qualitative descriptive study

**DOI:** 10.1186/s12913-023-09617-y

**Published:** 2023-06-09

**Authors:** Rebecca Clark, Jessica Gaber, Julie Datta, Samina Talat, Sivan Bomze, Sarah Marentette-Brown, Cherie Gagnon, Doug Oliver, Larkin Lamarche, Pamela Forsyth, Tracey Carr, David Price, Dee Mangin

**Affiliations:** 1 McMaster University, 100 Main Street West, L8P 1H6 Hamilton, ON Canada; 2 Canadian Red Cross, 5700 Cancross Court, Mississauga, ON L5R 3E9 Canada; 3 Windsor-Essex Compassion Care Community, 6038 Empress Street, N8T 1B5 Windsor, ON Canada; 4 University of Otago Christchurch, Christchurch, New Zealand

**Keywords:** Implementation science, Primary health care, Community-academic partnerships, Program implementation, Qualitative

## Abstract

**Background:**

Community-academic partnerships (CAPs) can improve the relevance, sustainability, and uptake of new innovations within the community. However, little is known about what topics CAPs focus on and how their discussions and decisions impact implementation at ground level. The objectives of this study were to better understand the activities and learnings from implementation of a complex health intervention by a CAP at the planner/decision-maker level, and how that compared to experiences implementing the program at local sites.

**Methods:**

The intervention, Health TAPESTRY, was implemented by a nine-partner CAP including academic, charitable organizations, and primary care practices. Meeting minutes were analyzed using qualitative description, latent content analysis, and a member check with key implementors. An open-answer survey about the best and worst elements of the program was completed by clients and health care providers and analyzed using thematic analysis.

**Results:**

In total, 128 meeting minutes were analyzed, 278 providers and clients completed the survey, and six people participated in the member check. Prominent topics of discussion categories from the meeting minutes were: primary care sites, volunteer coordination, volunteer experience, internal and external connections, and sustainability and scalability. Clients liked that they learned new things and gained awareness of community programs, but did not like the volunteer visit length. Clinicians liked the regular interprofessional team meetings but found the program time-consuming.

**Conclusions:**

An important learning was about who had “voice” at the planner/decision-maker level: many of the topics discussed in meeting minutes were not identified as issues or lasting impacts by clients or providers; this may be due to differing roles and needs, but may also identify a gap. Overall, we identified three phases that could serve as a guide for other CAPs: Phase (1) recruitment, financial support, and data ownership; Phase (2) considerations for modifications and adaptations; Phase (3) active input and reflection.

## Background

Collaborative relationships between community organizations and academic institutions, often known as community-academic partnerships (CAPs), are an effective way to improve the relevance and sustainability of research studies and the uptake of evidence into practice [1]. Having community partners involved in research facilitates the development of relevant research questions and outcomes, uptake of information into practice, and the exploration of the feasibility and sustainability of new innovations [1, 2]. All partners within a CAP benefit from knowledge exchange, access to each other’s resources, and the potential for future collaborations [1, 3–5]. CAPs are used in many contexts, including health care, and have been successful with health promotion and screening, disease management, health education, and other areas [1, 4, 6, 7]. While CAPs have the potential to improve research quality, collaborations that include multiple individuals and organizations also have the potential to become unwieldy, with multiple perspectives on each decision. Frequently cited barriers to CAPs include: unclear roles and expectations of each partner; financial pressures; different organizational cultures, processes, and terminology; having time to participate; different views on timelines; unequal power dynamics; and mistrust [1, 3, 6, 8, 9].

Health TAPESTRY is a community program centered in primary care which helps older adults stay healthier for longer where they live; the Health TAPESTRY model has four key elements: volunteers, interprofessional health care teams, technology, and community engagement and connections [10, 11]. In the intervention, community volunteers visit clients in their homes and administer health-related surveys and ask about clients’ goals using a web-based application on a tablet (TAP-App). Client responses are sent as a report to their primary care team where an interprofessional group of health care providers (the “huddle”) meets to view the report from their own professional lenses and offer further support and follow-up [10, 11]. We evaluated this implementation scale-up of Health TAPESTRY using the Reach, Effectiveness, Adoption, Implementation, and Maintenance (RE-AIM) framework which included a multi-site randomized controlled trial (RCT) that aimed to assess the reproducibility of the results from the first implementation, and the feasibility of scaling up the program in six sites across Ontario, Canada [10, 11]. The results of the full evaluation using the RE-AIM framework are reported in full elsewhere [12]. In this paper, we discuss the CAP as part of the implementation scale-up of Health TAPESTRY.

To implement Health TAPESTRY, a CAP was created between an academic institution, two non-profit community organizations, and six primary care clinics. Collaborating between nine partners while implementing a multi-site RCT was a large undertaking. While we used the RE-AIM framework [13] combined with Normalization Process Theory (NPT) [14] to understand the overall implementation of Health TAPESTRY and the associated RCT [12], these frameworks have both broader and different foci than would be needed to understand how the differing groups collaborated to implement Health TAPESTRY. Currently, little is known about what CAP partners’ focus on and discuss during implementation and how that relates to the overall implementation and perception of a program’s success. With many individuals involved, decision making and collaboration at the partner level are bound to impact both the individuals implementing the program daily and program clients. The Consolidated Framework for Implementation Research (CFIR) has 37 constructs in five domains: innovation (about the program being implemented), outer setting (beyond the implementation setting), inner setting (within the implementation setting), individuals (their roles and characteristics), and implementation process. [15] CFIR offers a broader picture of implementation that allows the opportunity to compare to partnership work such as this, and so we used it in this manuscript to help organize our results. This paper has two aims: first, to better understand and articulate the activities and the learnings from implementation of this complex health intervention by a nine-partner CAP at the planner/decision-maker level, and second, to explore how that relates to the experiences of those implementing the program at local sites.

## Methods

### Study design and setting

We used a qualitative descriptive approach [16] to describe the phenomena we were seeing in the transcripts alongside latent qualitative content analysis to go deeper into the meaning of those phenomena, based on the perspective of researchers who were themselves embedded in this implementation [17]. Health TAPESTRY was implemented in six communities across Ontario, Canada with clients who were patients of six Family Health Teams (FHTs). FHTs are primary care practices where physicians are formally connected to other interprofessional providers (e.g., nurses, dietitians, pharmacists, occupational therapists) to improve health care services [18]. This study was reviewed and approved by the Hamilton Integrated Research Ethics Board (#3967).

### The community academic partnership

The CAP in this study had four different types of partners: an academic family medicine department of a university (McMaster University Department of Family Medicine) a national humanitarian charitable organization (Canadian Red Cross) a local community coalition (Windsor-Essex Compassion Care Community) and six primary care practices (all FHTs) across Ontario, Canada [11]. The national charitable organization and the local coalition (henceforth termed volunteer organizations) were approached by the academic partner to help develop and manage the volunteer program, which is a core element of Health TAPESTRY [19]. The primary care practices were approached by the academic partner through existing contacts, other than one primary care practice which approached the academic partner based on knowledge of the previous implementation of Health TAPESTRY.

Multiple formal collaborative membership agreements were created for the CAP. The agreements were either three-party or four-party agreements made between the academic partner, the volunteer organization, and a primary care site (in one case, two primary care sites were partnered under the same agreement). The agreements outlined: benefits to the primary care practice; commitment and service requirements of each partner; financial arrangements; confidentiality; data management and sharing; and branding guidelines. The three-year study was funded through a provincial government grant and a private donor.

There were two groups that actively met to provide oversight for the program and research study. A general governance group met a few times a year and was responsible for the overarching governance of Health TAPESTRY. This group maintained the integrity of the four main elements of the program and explored the potential scale-up of Health TAPESTRY. It consisted of individuals from the academic partner (e.g., department leaders, researchers), and key leaders from the national charitable organization. The second group met monthly and was responsible for determining regular needs in the operational implementation of the program (e.g., solving problems that arose). Group membership was very similar to the governance group with the governance group being slightly larger including some additional members from the academic partner. Additionally, there were quarterly telephone meetings which we termed ‘community of practice’ meetings, where all partners were invited to attend, including select volunteers and health care providers from each site. The meetings were opportunities for the sites to connect with each other, and discuss progress and successes, as well as tod problem solve any barriers partners were experiencing. Lastly, there were bi-weekly meetings between the academic partner and the Volunteer Coordinators from the two volunteer organizations where all partners provided updates, solved any issues that arose, and helped the communities maintain fidelity to the program.

### Data collection and analysis

Data included meeting minutes and a survey of clinician and client stakeholders. A specific framework was not used to guide the analysis; however, our intention was to try and understand the sensemaking work (or coherence) that the CAP partners, implementors, and clients engaged in when implementing Health TAPESTRY [14]. After we analyzed both data sources, we compared and contrasted the categories in both data sources to determine where differences and overlap of topics occurred, and then also compared these topics to the domains in CFIR [15] to allow for easier comparison to other programs.

### Meeting minutes

We conducted an a priori analysis of meeting minutes using qualitative descriptive analysis and latent content analysis (as described in “Study Design and Setting”) to make sense of the implementation of Health TAPESTRY. Meeting minutes were collected from all meeting groups listed above as well as meetings between the academic partner and primary care teams or volunteer organizations. All meeting attendees provided informed written consent to the meeting minutes being analyzed.

We used Bengtsson (2016)’s four stages of qualitative content analysis to analyze the meeting minutes [17]. We began with Stage 1, Decontextualizing, wherein two members of the research team (RC and JG) independently coded the dataset; Stage 2, Recontextualization was done both after and alongside this stage, where we continuously moved between the two stages to identify individual units of meaning while also aiming to understand those smaller units of meaning within a transcript or the dataset as a whole. While the meeting minutes were inductively coded, the two coders were deeply embedded in the project, both involved in the implementation and evaluation of the program, and one of the two also an attendee at many of the meetings in the dataset. This meant that while there was no coding tree or framework developed a priori, we coded based on our knowledge of Health TAPESTRY including its four key elements (volunteers, interprofessional health care teams, technology, and community engagement and connections). All coding was completed using NVivo 12 [20]. Once all meeting minutes were coded, we began Stage 3, Categorization, where the two coders met to discuss the codes and group similar codes into categories. We chose to use and present categories rather than themes as we were more interested in the topic of discussion rather than the specific exemplar, and more interested in generalizability over specificity based either on context or individual speaker [21]. The criteria for creating these categories were whether they were salient topics of discussion across the meeting minute dataset that had to do with the implementation of Health TAPESTRY. This was latent rather than manifest content analysis as the coders were able to use their understanding of Health TAPESTRY to understand what speakers *intended* to say [17].

To further make sense of the meeting minute data, after initial analysis we completed a member check with selected participants who had been active members of the meetings and in the implementation of Health TAPESTRY. Member checking is commonly used in qualitative research to validate and assess the credibility of the data [22–24]. The purpose of our member check was to determine if the results of the meeting minute analysis resonated with the participants’ experience, and to gain further thoughts on the process of implementing Health TAPESTRY that were not represented by the minutes alone. We invited representatives of the academic partner, both volunteer organizations, and one primary care practice to participate. We conducted one focus group and one interview (due to scheduling constraints). Prior to the focus group/interview, participants received a copy of the meeting minute analysis results to familiarize themselves with the content. During the sessions, two co-facilitators (RC and JG) presented the meeting minute analysis results, and then facilitated a semi-structured discussion. The facilitators took field notes.

### Survey

Clinical team members and clients were asked about the five best and five worst elements of Health TAPESTRY in an open-ended survey. These were not ranked responses. Clinical team members were asked this when they participated in focus groups, and almost all responded on paper, though some sent virtual responses outside of the focus group time. Clients were asked this in their six-month volunteer visit, and volunteers entered their responses into the TAP-App. The volunteers were not asked to complete this survey.

The best/worst survey data were qualitatively analyzed using Braun and Clarke’s six steps of thematic analysis [25] but at a more semantic rather than reflexive level (i.e., representing what was said rather than representing underlying ideas through researcher reflexivity or introspection). After familiarizing themselves with the data (step 1), three reviewers (RC, SD, and JG) generated initial codes from the data set (step 2) independently without any a priori coding structure. The reviewers then worked together to search for themes (step 3), review themes (step 4), and define and name themes (step 5). This work was conducted using NVivo 12 [20]. As the survey was about the Five Best and Five Worst elements of Health TAPESTRY, we chose to report only the five most salient (i.e., common) themes from each aspect (best/worst) by each of the groups (clients/providers) when we reported on our findings (step 6) in this paper.

## Results

### Participants and sources

We analyzed 128 documents for the meeting minute analysis. We invited six people from three stakeholder groups to complete the member check and all six agreed (four people from the volunteer organizations, one person from a primary care practice, and one person from the academic partner).

The survey was completed by 49 health care team members and 228 clients. See Table 1 for their demographics.

**Table 1:** Demographics of Survey Participants

Variable	Health Care Providers n = 49	Health TAPESTRY Clients *n = 228*
Gender*		
Man	11	80
Woman	38	146
No response	0	2
Age (years)		
Range	Not collected for this group.	70–97 (n = 227)
Mean (SD)		77.07 (5.37)
Years of experience in primary care (for providers)		
Range	1–51 (n = 42)	Not collected for this group.
Mean (SD)	9.55 (10.79)	
Professional role (providers)		
Physician	20	Not collected for this group.
Nurse (RN/RPN)	6	
Occupational Therapist	4	
Director/Manager	4	
Dietitian	3	
Nurse Practitioner	3	
Social Worker	3	
Administrative Assistant	2	
Pharmacist	1	
Physician Assistant	1	
Physiotherapist	1	
System Navigator	1	

*While the questionnaire included non-binary gender options, there were no responses in these categories; only categories where there are data are included in this table.

### Meeting minute analysis and member check

During meetings, we found that the CAP partners focused on the following categories: primary care sites; volunteer coordination; volunteer experiences; internal and external connections; and sustainability and scalability. These key categories mirror the four key elements of Health TAPESTRY (volunteers, interprofessional health care teams, technology, and community engagement and connections), other than the technology element, which was not a prominent topic of discussion in the meetings included in the dataset. The sub-topics discussed in each category included a focus on barriers or challenges, as well as general updates on study progress, feedback, and positive impacts of Health TAPESTRY. Further detail for each category is below. In Table 2, we also match the categories and sub-topics with an associated key element of Health TAPESTRY, to understand how it lines up with our intervention framework, and CFIR [15] domain and construct, to facilitate comparison of this work to interventions beyond Health TAPESTRY.

**Table 2:** Meeting Minute Data Mapped to Health TAPESTRY Elements and CFIR Domains

Category and Key Sub-Topics from This Dataset	Health TAPESTRY Element	CFIR Domain (Construct)
Primary Care Sites • Barriers to site onboarding • Facilitators to site onboarding • Adaptations between sites • Barriers to program implementation at local sites • Strategies to improve implementation at local sites	Interprofessional health care teams	Barriers and facilitators to onboarding: • Process (Engaging) • Inner Setting (Implementation Climate, Readiness for Implementation) Adaptations between sites: • Intervention Characteristics (Adaptability) Barriers and strategies for program implementation: • Inner Setting (Culture, Implementation Climate, Structural Characteristics, Networks and Communication) • Intervention Characteristics (Adaptability, Complexity) • Outer Setting (Patient Needs & Resources) • Characteristics of Individuals (Knowledge and Beliefs about the Intervention)
Volunteer Coordination • Volunteer recruitment • Barriers to volunteer program implementation • Strategies to facilitate volunteer program implementation • Navigating collaboration and partnerships	Volunteers	Recruitment: • Process (Engaging) • Intervention Characteristics (Adaptability) Barriers and strategies: • Process (Engaging, Executing) Navigating collaboration: • Outer Setting (Cosmopolitanism) • Inner Setting (Networks and Communication, Structural Characteristics, Culture)
Volunteer Experiences • Volunteer experiences	Volunteers	• Characteristics of Individuals (Knowledge and Beliefs about the Intervention, Self-efficacy, Other Personal Attributes) • Intervention Characteristics (Complexity)
Internal and External Connections • Internal program connections between clinical team and volunteer organizations • Connections beyond the program	Community engagement and connections	Internal program connections: • Inner Setting (Structural Characteristics, Networks and Communication, Culture, Implementation Climate) Beyond the program: • Outer Setting (Cosmopolitanism, Patient Needs and Resources, External Policy and Incentives)
Sustainability and Scalability • Possibility of continuation of the program	(Not a program element, but a topic related to program continuation)	• Inner Setting (Implementation Climate, Readiness for Implementation) • Process (Executing, Reflecting/Evaluating)

#### Primary care sites

When discussing the recruitment and onboarding of potential new Health TAPESTRY sites (i.e., the primary care organizations), conversations included reviewing the questions and concerns potential sites had about the program, and how to respond. The questions spread over several topics including recruitment, financial support and ownership of data. Further, the CAP identified facilitators to the site recruitment sessions, including the importance of having face-to-face conversations with the primary care practices. These topics (recruitment, financial support, and ownership of data) did not prominently appear in later data after this initial clarification, suggesting CAPs’ functions go through phases related to the time course of project implementation. This phase was also largely carried out by the academic and volunteer organization partners of the CAP, rather than the primary care teams which entered into the partnership later (as some of these discussions were about recruiting these clinical teams).

Once the onboarding phase was complete, the meetings discussed adaptations of Health TAPESTRY in each of the six communities (e.g., use of a template in clients’ electronic medical record, preparing packages of resources for clients), with the individuality of the distinct sites influencing the conversation needs of the CAP planners/decision-makers. Through the member checks we found that meeting minutes alone were not able to describe the individuality of each of the six primary care sites, though it was clear that differences between sites was a major area of discussion. Our CAP’s function through this next period moved to explicit consideration and problem solving for modifications and adaptations for different contexts and sites as outlined in detail below.

Barriers to the implementation of Health TAPESTRY relating to the primary care context included the difficulty of engaging health care providers (particularly family physicians) in the program, the frustrating process of client recruitment, logistical issues with the interprofessional primary care team that meets to review client information (the “huddle”), and providers not getting all the information they would like (e.g., getting inappropriate goals from clients). It is important to note that these barriers were not necessarily present in every site. Some of the specific client recruitment challenges discussed included clients having privacy concerns, volunteer coordinators being unable to reach clients who had been referred to the program, a lack of physician buy-in to refer clients, confusing recruitment materials, and clients not feeling that they needed the program.

The planner/decision-maker partners represented in the meeting minutes suggested some strategies which impacted implementation at the local sites. To facilitate recruitment they suggested: patient-facing materials to accompany the invite letter, physicians recommending clients, and large mailout efforts by the primary care practices. To improve implementation, they suggested: better huddle coordination (improving scheduling, having the Volunteer Coordinator present, having the family physician present, and having paper questionnaires on hand), and having engaged and active leadership for Health TAPESTRY at each clinical site (including a practice model champion and strong huddle lead).

#### Volunteer coordination

A key area of discussion related to volunteer coordination was volunteer recruitment. The volunteer organizations did a variety of advertisement campaigns to recruit volunteers for Health TAPESTRY (e.g., newspaper ads, Facebook, radio, paper materials, website postings). Different strategies were considered successful in different communities, and these conversations held at the decision-maker/planner level had to be altered for each context by the local volunteer coordinators. Also pertaining to volunteer coordination on the ground in local sites, there were discussions around strategies to help facilitate the volunteer program such as assistance with booking client appointments during peak recruitment.

Barriers identified through our meeting minute analysis pertaining to the implementation of the volunteer program within Health TAPESTRY included difficulties recruiting volunteers, limited volunteer availability, and the long period of time between volunteers receiving training and being able to have their first meeting with clients. Through member checking, difficulties scheduling client visits due to competing schedules was identified as an additional barrier not captured through the meeting minute analysis. These barriers were identified both directly by volunteer coordinators situated in local implementation sites, and the organizations they were a part of at the decision-maker/planner level.

From a partnership-specific standpoint, there were also discussions relating to navigating collaboration and partnerships between the academic partner and volunteer organizations. These discussions included: clarifying how best to collect data for research purposes; clarifying which partner the data in the study “belongs” to (the academic partner, as research lead) and whether the volunteer organizations could use it for their purposes (only in certain, de-identified scenarios); branding and communications, including the decision to co-brand all products with both the academic partner and volunteer organizations’ logos; and preparation of an agreement to formalize the partnership between the legal arms of the academic partner and the volunteer organizations.

#### Volunteer experiences

Based on perspectives by volunteer coordinators and the volunteer organization partners, volunteers faced challenges during their participation with Health TAPESTRY such as gaps in knowledge (e.g., needed more information on community resources), issues with surveys, technology difficulties, and transportation issues when attending client visits. There were also discussions about reasons why volunteers withdrew from the program and potential strategies that could facilitate the volunteer program.

#### Internal and external connections

During the program there were issues regarding the connection, or lack thereof, between the clinical teams and volunteers. This information filtered up from the partners implementing the program at local sites and was dealt with quite extensively at all levels of meetings. Clinical teams were not utilizing the volunteers to their full capacity and volunteers wanted to be more fully engaged in follow-up appointments with clients and feel more connected to the clinic. Prior to the program though, the clinical teams had been enthusiastic about the volunteers and their potential.

Other aspects of this internal and external connections theme included discussion about additional surveys or related projects (both internal and external to the CAP) that were explored, but ultimately were not implemented within Health TAPESTRY. These additional surveys and projects had varied sources from local project implementers considering adding a theme to the surveys, to investigators from the academic team considering adding an element to evaluate; ultimately, wherever their source, these additional ideas tended to be considered and abandoned due to feasibility, interest by other implementers, or appropriateness for connecting to this program or its client population.

#### Sustainability and scalability

A noticeable later function of our CAP was a focus on active input and reflection. This is seen in the discussion and its focus on sustainability and scalability. There were discussions about the possibility and feasibility of Health TAPESTRY being implemented in each site as a program with no research element present; in some sites these started from the implementer level, as they wanted to be able to continue the program. In other sites, however, this was a top-down discussion only. Specific topics of discussion around sustainability and scalability at the decision-maker/planner level included the interest of, and capacity at, each site to continue, possible client recruitment strategies (including improved messaging for clients to understand the benefits of the program), and the necessity of multiple partners to align. Knowledge translation strategies during the research study included community of practice meetings and events, videos, and sharing patient stories.

#### Overall views on program

There was general positive feedback from the clinical sites about the value of Health TAPESTRY and the ease of implementation. Health care providers felt that the program had value, was beneficial for clients and their team, and was easy to implement. They also felt that engaging volunteers to do home visits was appreciated, and they learned new things about the clients as well as about community resources and services.

### Survey: best and worst elements of health TAPESTRY

The survey makes visible some key areas of significance and concern to the clinic site members of the CAP, as well as the clients of the program. Table 3 shows the best and worst elements of Health TAPESTRY according to the interprofessional health care team members as well as clients. A large number of clients reported there was nothing bad about Health TAPESTRY; in this table we report only on those who responded with something specific.

**Table 3:** The five best and the five worst elements of Health TAPESTRY.

Five Best Elements		
Clients (N = 228)	Interprofessional Team Members (N = 50)	
They gained knowledge and awareness about programs, services, and resources available at the clinics or in the community	The regular interprofessional team discussions	
They enjoyed socializing with people in their own homes	The comprehensive picture of the clients	
It contributed to increased reflection on and awareness of their health goals and general self	The collaborative approach that connected and strengthened the interprofessional team	
The clinical follow-up	Clients were connected to additional supports with their care team and community	
The volunteers and other people involved with Health TAPESTRY were helpful, respectful, and good listeners	a) The program identified people at risk and their needs* b) The program engaged clients with their care team and their own health care*	
**Five Worst Elements**		
The volunteer visits and surveys took too long to complete	Being involved was time consuming	
The survey questions were redundant, repetitive, and confusing	There are limitations with the issues and goals that Health TAPESTRY can address	
There was a lack of or delay in follow-up	Clients were confused about Health TAPESTRY or their plan of care	
They felt the program did not help them	It was unclear how follow-up occurs and who is responsible	
They did not understand the main objectives of Health TAPESTRY	Clients did not follow through on interprofessional team recommendations	

*These responses had an equal number of codes; Participants could give more than one response for the best and worst

Clients and interprofessional team members both found strengths in the program in the aspects they specifically interacted with (for clients, this was volunteer home visits, and for providers, this included the regular interprofessional “huddle” discussions). They both also spoke of connection, collaboration, and engagement with other people and other services as a positive. There were also mirrors in what they found were the “worst” parts in terms of time involved in the intervention and confusion over the elements they were involved in. They also both spoke of efficacy in these top five worst areas, but clients who mentioned this just said the program did not work for them, while providers asid that clients did not follow through We noted that when these primary care teams and clients “on the ground” during the implementation of Health TAPESTRY were asked the best and worst aspects of the program, most of the ideas raised about the best aspects of the program and many of the issues were not discussed at the planner/decision-maker level. There were differences between what partners at the planner/decision-maker level focused on when implementing an intervention, which were largely around ensuring the implementation happened, and what elements or issues that the people implementing the program day to day and the clients receiving the program noted. For example, most of the ideas raised around the best aspects of the program were not discussed at the CAP level.

## Discussion

The nine partners from the planner/decision-maker level of the CAP discussed a breadth of topics in meetings throughout the preparation for and implementation of Health TAPESTRY. Given the complexity of the intervention and number of partners, these findings are unsurprising. Downstream from the overarching CAP, in the survey, clients and health care team members also identified several areas of significant positive impact, as well as areas of concern that relate to a wide variety of program areas. It is useful to note that some of these overlapped with discussions by the planner/decision-maker CAP partners, however some did not. Through our analysis, it was evident that the CAP in this study did experience some of the challenges that have been reported by other scholars on research on CAPs. These included unclear roles, time commitment, and unequal power dynamics; [1, 2, 6] however, these concepts were largely represented in the survey data from the people involved “on the ground” at local sites rather than in the meeting minutes from program planners/decision-makers. It was health care providers implementing the intervention and clients receiving it who most noticed the unclear roles and burden of time commitment of program participation, and the power dynamics were shown by the differences in focus between the clients/providers on the ground and in the topics arising in meetings. Unlike other research studies [1, 3, 6, 8], we did not find some of the common issues such as mistrust, financial pressures, different organizational cultures, processes, or terminology, although terminology was discussed. Further, based on views from health care providers at local sites, the program was valuable for the clients and relatively easy to implement, indicating that the CAP and its discussions had a measure of success; overall the CAP benefited from the collaborative efforts of all partners to successfully implement the program.

An important area of learning is around the areas and stakeholders that had “voice” at the CAP level. When primary care teams and clients – the individuals who were “on the ground” during the implementation of Health TAPESTRY – were asked the best aspects of the program, most of the ideas raised were not discussed at the CAP level. For example, clients identified socializing with the volunteers in their homes to be a highlight of the program, which was not discussed by CAP partners. Clients also noted how helpful and respectful the individuals involved in delivering the program were. It could be surmised that the CAP partners did not discuss these topics for two reasons. First, meetings at the partner level were focused on the program’s high-level protocol, issues that arose, or major problems to be solved whereas both the home visits and the recruitment of strong volunteer teams were already accepted hallmarks of the program from the first implementation. Second, the CAP partners did not heavily, or at all, discuss more minor issues, problems that arose for the on-the-ground implementors, or things unrelated to the protocol. For example, individuals being respectful and helpful was more a product of the people involved and less about the program’s protocol, which would have made it difficult to discuss at the CAP partner level. This may also explain the fact that of the four key elements of the Health TAPESTRY model (volunteers, interprofessional healthcare teams, technology, and community connections and engagement), technology was not heavily discussed at the planning/decision-maker level of the CAP.

When we compared the topics discussed in the meeting minutes with an established framework for implementation, CFIR [15], we found that of the five constructs of CFIR, the discussions at the planner/decision-maker level of our CAP focused particularly on the Inner Setting (which included both clinical teams and volunteers). The constructs of Outer Setting and Process were also well represented, with only slim representation from the final two constructs, Intervention Characteristics (other than *adaptability*, a key domain seen in our data) and Characteristics of Individuals. At one level this makes sense as individuals from the planner/decision-maker level of the CAP were the source of the intervention, so would not need to discuss these details of the intervention that had been established in previous research; characteristics of individuals is an attribute that would be more relevant with the people implementing the project at local sites. The lack of representation of some of the clinician and client perspectives we identified in the survey in the higher-level CAP discussions indicate a value for considering a process for these “voices” to feed in to the CAP discussions during implementation. For example, while the survey results of the best and worst elements of Health TAPESTRY may have indicated that clients and providers involved in local site implementation may have been considering how the program became “normalized” into normal practice, such as is seen in Normalization Process Theory [14], the meetings of the planner/decision-maker level of the CAP were more concerned with the often earlier and more concrete elements of ensuring the program implementation happened.

We identified three phases in the process of this CAP that could serve as a guide for explicit planning for CAP process and agenda setting. In Phase 1, recruitment, financial support, and ownership of data were issues that required discussion and clarification. It might also be useful to identify at this phase what each organization hopes to gain from the project, as this was an area that the surveys suggested was unclear at times. Phase 2 is where explicit consideration and problem solving for modifications and adaptations for different contexts and sites should occur. Phase 3 is about active input and reflection on process experiences of planning/decision-making partners and integration of experiences of those involved in direct implementation at local sites (e.g., volunteers and clinicians). We also noted that three of the four key elements of the Health TAPESTRY intervention were mirrored in the key category areas in our analysis. This suggests that key intervention elements could be included in CAP processes explicitly, particularly in Phases 2 and 3 but also in the preparation phase.

### Implications for future practice

Based on the implementation of Health TAPESTRY using a CAP, we have three key learnings and propose suggestions for future work. First, we did not actively engage clients (a stakeholder group) in the design or implementation of Health TAPESTRY. Instead implementors relied on qualitative feedback (from the previous and current implementation) to address client concerns. Considering the number of barriers clients communicated, the discussions that were directly related to the clients, and that stakeholders can engage in knowledge exchange from each other, among other advantages [1, 3–5], we believe more active engagement would have been beneficial.

Second, due to the objectives (i.e., reproduce the effectiveness of the first implementation of Health TAPESTRY) and design of the study (i.e., RCT), the acquisition of funding, and the performance requirement not all partners had equal influence regarding study decisions. The academic partner in the CAP did retain a large amount of control in certain aspects of the study (e.g., protocol requirements critical to the study’s integrity and grounded in ethic board approval), despite good intentions. Other authors have also reported unequal power dynamics [2, 26]. The national charitable organization also had more influence compared to the other partners due to their continuous in-depth involvement in the volunteer program and committees throughout the program. Based on negative issues raised by the health care providers, we suspect that the power dynamics and protocol constraints could partially explain our findings, despite the goal of an equitable CAP. For example, if the primary care teams felt more empowered to make decisions about the program, they may have had greater clarity regarding workload and who was responsible for providing follow up care to clients. This finding aligns with unclear roles being a commonly cited barrier in CAPs [1]. We urge future CAPs to thoroughly discuss early on the core aspects of the research that should not be altered, and what domains other partners have full control over. This should help to develop more clear and equitable partnerships, which are important to the sustainability of the CAP [6]..

Lastly, we encourage future CAPs to assess discussions and feedback from multiple levels of program implementation to determine where discrepancies and agreement exist on different elements of a program. For example, in this project, community involvement and engagement are important aspects of Health TAPESTRY as they were a topic of discussion by the CAP as an area for improvement, as well as identified by both clients and health care providers as a positive element of the study. Therefore, a further exploration and development of important program elements such as community involvement should be considered prior to implementation.

This study had strengths and weaknesses. A prominent strength of this study was the large and varied sample as well as using the member check process with key implementation stakeholders, which allowed us to validate the implementation story and gain final reflections on the program’s progress. Second, the structure and organization of this large CAP seemed to contribute to a streamlined decision-making process that avoided unresolved issues and other common barriers such as financial pressures and differing timelines even with many CAP members. A limitation of the study was that the meeting minute analysis and member check were not planned a priori, which meant that some meeting minutes lack detail that would have benefitted this analysis. A second limitation, due to the nature of the study and number of partners, is that many decisions were made by email or through informal conversations, which were not captured. Therefore, our analysis likely missed topics and decisions. Lastly, the member check discussion was not anonymous and was facilitated by the academic partner. Therefore, participant responses may have been biased as to not offend other partners in the CAP.

## Conclusion

Using the CFIR was useful in identifying general areas for guiding optimization of CAP process. The specific focus in this study on the experience of implementing a CAP provided insights and reinforced the need for learning not just from the outcomes of a program but from different aspects of implementation. The results of this study provided interesting insights into the differences between what CAP partners focus on when implementing an intervention and what elements or issues that the people implementing the program day to day and the clients receiving the program recall. Even though the CAP was essential to the implementation of Health TAPESTRY, some of the lasting impacts of the program identified by clients and health care providers were not discussed at the partner level, potentially because they were already accepted elements of the program or more on-the-ground protocol issues that did not require CAP level discussion. Our findings identified several distinct phases as well as broad context areas in the process of CAP implementation and these could be used as a guide to optimize CAP process. Our findings also highlight the importance of gaining feedback and insights from implementors at multiple levels, and a process for integrating these voices, as there will likely be differing opinions on the elements of implementation as well as the significant value of equitable partnerships within a CAP to facilitate implementation.

## Data Availability

De-identified data can be available upon a reasonable request to the corresponding author.

## Abbreviations

CAP: Community academic partnership
FHT: Family Health Teams
